# Dr. Seymour on Ovarian Dropsy

**Published:** 1830-04-01

**Authors:** 


Illustrations of some of the Principal Diseases of the Ovaria, their Symptoms and Treatment: to which are prefixed, Observations on the Structure and Functions of these Parts in the Human Being and in Animals. By *Edward J. Seymour*, M.D. &c. &c. Physician to St. George's Hospital. 8vo. pp. 128, with 14 Lithographic Plates, 1830.

Dr. Seymour deserves great praise for the zeal with which he prosecutes the study of morbid changes in the human body, and the present work affords ample proof of the talent which accompanies and directs that zeal.

In the first chapter of the work Dr. Seymour gives a concise but clear sketch of the structure and functions of the ovaria, first in the human subject, and then in various animals. This chapter displays great research, and much ingenious reasoning. It would be impossible for us to do it justice by extract or analysis, since it is, in itself, a close analysis and critique. But we recommend it to the attentive perusal of the reader. One passage only, near the end of the chapter, we shall here introduce, as it contains Dr. Seymour's conclusions respecting the long disputed subject of Corpora Lutea.

"It must be admitted by all that corpora lutea exist without impregnation occasionally. They are figured by Valisneri and Sir E. Home; and the observation has been confirmed by many living physicians and surgeons. That it occurs after impregnation, is certain, and proved by the observations of Haller, who traced their gradual formation; but if, as supposed by Sir E. Home, they are necessary to render the ovum fit for impregnation, they should exist nearly always in virgin animals at the time of puberty. This is by no means the case. It has occurred to me to have examined the ovaria in the human being, and in animals at the period of puberty, in very many instances; many had ova ready for impregnation, large, projecting, vascular, yet no corpora lutea were visible, which induces the following conclusion, that in every instance these animals must have been barren, or that the formation of corpora lutea is not a necessary preliminary process to impregnation.

"From these premises, comparisons, and observations, my opinion has been formed, that corpora lutea are the result of the change which takes place in the ovarium by the bursting and discharge of the ovum, occurring rarely in virgin animals, because the bursting of the ovum is not a frequent but only possible occurrence, but always following impregnation, and diminishing as gestation proceeds.

"It may here be asked, of what advantage is it to determine accurately the formation of these bodies? We have seen that their production is probably influenced by strong moral as well as physical impressions, the result of great vascular excitement of the part, and their absorption effected by great activity in the vessels of that system. Any deficiency, then, in the quantity of vascular excitement necessary—any obstacle to the exercise of absorption—would produce changes in these parts differing from the natural ones, which they were intended to undergo,—would, in a word, produce disease; and it remains to be discovered whether any of the serious and complicated diseases of these organs are to be traced to alterations which the corpora lutea undergo from any or all of these causes." 33.

Seven beautifully executed plates illustrate the anatomy and physiology of the ovarian organs, comparative and human.

In the 2d chapter Dr. Seymour enters on the subject of structural diseases of the ovaria. These diseases are arranged under three heads—those arising from inflammation—those arising from enlargement of the natural structure, and those from addition of new structure formed by disease:—these last including scirrhous and fungoid growths—lastly, those deviations from natural structure which result from obstruction of function, and also congenital malformations.

"Inflammation of an acute form attacks the substance of the ovarium, which has been found in a state of suppuration after acute inflammation of the womb and its appendages in women who have died in child-bed. This likewise does not appear to be marked by any peculiar symptom: the suppuration in such cases has been of the diffuse kind.

"Softening also takes place as the result of acute inflammation of these parts. A case recently occurred under my observation, where death from inflammation of the womb occurred about three days after delivery. The whole of the cellular membrane under the peritoneal covering of the uterus, and under that lining the pelvis, was in a state of diffuse suppuration, and the absorbent vessels loaded with pus could be traced nearly as high as the diaphragm. The ovaria were in a state of extreme softness, presenting the appearance of a vascular pulp, but no purulent matter was visible.

"The substance of the ovarium is likewise subject to inflammation of a chronic form, which may certainly exist independently of inflammation of the substance of the uterus or its coverings. Abscess of the ovarium does indeed appear to be a rare disease, but it nevertheless occurs; and indeed, in reasoning on the subject, it would not be easy to account for the difficulty or impossibility of inflammation and its result, suppuration, occurring in the loose cellular texture of this organ. The following case of this disease will best describe the symptoms and post-mortem appearances.

"A young woman, aged 17, of the lowest and most unfortunate class of females, was a patient of Guy's Hospital, under the care of Dr. Bright, in the autumn of 1823.

"She was greatly emaciated, had a very quick and feeble pulse, a shining red tongue, and constant watchfulness. She suffered from constant and irrepressible diarrhœa, and for many successive days vomited both food and medicine: the catamenia were absent. The case made a considerable impression on my mind, from the extreme emaciation and colliquative diarrhœa, without any evident symptoms of disease of the lungs or intestinal canal. After having been in the hospital about two months, she suddenly complained of most acute pain over the abdomen, and in a few hours expired.

"On opening the abdomen, death appeared to have been produced by the effusion of a large quantity of pus into the peritoneal cavity, which escaped from an abscess in the right ovarium, which abscess appeared to arise from suppuration in the substance of the viscus, similar in every respect to phlegmonous abscess in any part of the body, and not connected with any cyst or change, or addition of structure, the product of morbid growth." 40.

Chronic inflammation of the ovarium, like chronic inflammation elsewhere, ends in thickening and enlargement of the part—such cases often remaining stationary for many years. Dr. Seymour hazards some speculations on disease of the Graafian vesicles, or corpora lutea, as occasioned by excited feelings connected with the uterine system; and queries whether those instances of purulent depôts, found in cysts of the ovaria, might not be owing to some of the superficial vesicles having undergone rupture and change from the above sources of excitement, independent of pregnancy, and arrived at the purulent state described by some authors. The fluid of the Graafian vesicle is liable to disease. It is often red, and even black, from admixture of blood; and, from the following case, it appears to our author that it may become altered from imperfect fecundation.

"A woman, æt. 31 years, was admitted into St. George's Hospital in November last, labouring under ascites and anasarca, depending on the heart becoming enlarged after repeated attacks of rheumatic inflammation. By moderate blood-letting and ordinary diuretic medicines, combined with mercury, her dropsy entirely disappeared; and feeling no further inconvenience, she was dismissed at her own request, warned, however, that the smallest imprudence would bring back her disease. She remained at home a month, during which time she cohabited with her husband: the symptoms returned; she was admitted again, relieved, but died suddenly six weeks after her re-admission; a death explained by the enormous dilatation of the heart, and an aneurism of the substance of the left ventricle, immediately below the mitral valves. A very curious appearance was found in the right ovary; a collection of serous fluid, about the size of a large pea, contained in a delicate membrane, of an elongated oval form, was found arising from the coats of the ovarium; at its origin having a communication with the internal structure, and appearing exactly as if it had escaped from thence: to the other end the fimbriated extremity of the fallopian tube adhered. It appeared to me that a Graafian vesicle had burst; that, not having freed itself entirely from the coats of the ovarium, it could not pass into the fallopian tube, but remained embraced by that organ, and underwent a partial development." 44.

By far the most common form of ovarian disease, however, is the conversion of this organ into numerous cysts, which, when containing fluid, is termed ovarian dropsy. Under this denomination have been included simple serous cysts, formed in the broad ligaments and fallopian tubes.

"All these, confounded together under the name of hydatids, are distinguishable from the latter, by being nourished by vessels supplying them from the parts in which they are formed, vesicles to which the name hydatid is attached being nourished by their own blood-vessels, or, in other words, having an independent life. Occasionally one or both ovaria are converted into simple cysts; the whole of the cellular substance and vesicles disappearing, that which was the fibrous coat of the ovarium becoming the fibrous coat of the cyst." 45.

The first and simplest form of the disease, is an enlargement or alteration of the corpora Graafiana. At an advanced period of life, on cutting into the ovarium, one or more of the Graafian vesicles are found dilated, and these bodies, generally the size of a millet-seed, become as large as an almond—are filled with limpid fluid, and their internal membrane becomes very vascular. Occasionally they enlarge to a greater degree, and always on the side nearest the proper coat, which often becomes distended to an enormous size. In this way, it appears to Dr. Seymour that a large single cyst, with a fibrous covering, may be formed—and this, he thinks, is the simplest form of ovarian dropsy, the internal membrane secreting a prodigious quantity of fluid. The same opinion is entertained by M. Cruveilhier. One or two of the Graafian vesicles undergo this change, when the disease consists of one or two cysts filled with fluid.

"A married woman, æt. about 60, was admitted into St. George's Hospital in September, 1828, in order to undergo the operation of tapping for the third time in five years, rendered necessary in consequence of the sufferings she experienced from the pressure of the tumor. About sixteen pints of ropy albuminous fluid, of a chocolate colour from admixture of blood, were drawn off. The patient, whose health was much broken, did not rally after the operation; and she died, as is often the case, not from inflammation occurring after the operation, but with symptoms of exhaustion, a week from its performance.

On opening the body, a large fibrous cyst was visible, pushing forward the broad ligament as far as the fundus of the uterus; and on the opposite side expanding into a sac, which reached nearly to the epigastrium, and contained several pints of coffee-ground fluid. At the inferior part of this sac were the remains of the ovarium, very much shrivelled and imperfect on the surface internal to the cyst. It appears to me that this is a specimen of the cyst which I have endeavoured to describe; an enlarged vesicle, such as we often see in its earlier stage, pushing forward and gradually dilating the fibrous coat of the ovarium, the remainder of the ovarium remaining attached to the inferior portion of the cyst." 47.

It is to this form that the name encysted dropsy is strictly applicable, and is the disease which exists so many years without much distress, furnishing, by tapping, such wonderful quantities of fluid. Much solid fibrous structure is occasionally connected with these collections; but more of this hereafter.

"The ordinary symptoms attendant on ovarian dropsy are very various, and by no means severe, and are limited principally to the effects of pressure on neighbouring parts. Where the increase of the disease is slow, the patient often suffers no other inconvenience than from swelling of the leg on the side on which the tumour is largest, or from the unsightly bulk of the abdomen, which she is unable to conceal. Patients have lived in this manner thirty or forty years, with a very considerable enjoyment of the comforts of life, and even the pleasures of the world, the accumulation of fluid rendering it necessary from time to time to perform the operation of paracentesis. In cases of this kind, symptoms dependant on unusually rapid increase of bulk, or pressure on any particular organs in the abdomen, occur. Thus heartburn, vomiting, and purging, difficulty of passing urine, or violent and severe head-ache, are met with, which are entirely removed if the bulk of the tumour be reduced. There is a case now under the care of Mr. North, of Berkeley-street, where the patient has for many years been unable to pass her urine, except by the daily use of the catheter; and this appears to arise from the natural situation of the bladder being altered by pressure, and perhaps by the adhesion of the tumour." 49.

When both ovaria are diseased in this way, the catamenia are always absent—when only one is affected, they are irregular or defective.

"In many cases the diagnosis of this disease is sufficiently easy. Pain has been felt in either iliac region, succeeded by a tumour, which can be traced low into the pelvis, and the uterus is found on examination dragged upwards by the morbid growth. The history likewise assists us: it has followed miscarriage or delivery; at other times it occurs in fema'es where pregnancy is out of the question, or at a time of life when it is impossible, and yet where the unbroken health renders ascites a very improbable occurrence. Occasionally, however, independently of its complication with pregnancy, it is difficult to distinguish this disease from accretions of the peritoneum with effusion, and still more so from ascites, the result of visceral obstruction; often also it occurs together with ascites.

"It appears to me that the following is one cause of the mistake of ovarian tumours (in which some of the cysts, or the whole cavity, is filled with fluid, the parietes consisting of solid matter) for ascites with visceral obstruction. It often happens that the increase of the ovarium is slow at the commencement, and extends by a narrow neck into the abdomen before it is perceived: adhesions take place between it and the neighbouring parts, and from that time the increase of its growth is rapid. Hence, in some cases, the patient persists in having first perceived it in the right or left hypochondriac regions, and the solid portions give to the touch the feeling of enlargements of the spleen and liver, when, if ascites be also present, the combination is very perplexing." 50.

When ascites is present, a different feeling is communicated to the hand on striking the abdomen, whether in front or on the hypochondria. When in the recumbent posture, the fluid gravitates, in ascites, into the hypochondria and lumbar regions—in encysted dropsy, the fluctuation remains circumscribed. If there be much air in the intestines, as well as fluid in the peritoneal cavity, the fluctuation will be much more sensible in ovarian tumour than in ascites.

"And in more cases than one most experienced practitioners have been surprised at the flow of the albuminous dark-coloured fluid of encysted dropsy, during the operation of tapping, instead of the transparent serum of ascites, which the very sensible fluctuation had led them to expect. Indeed, on striking the abdomen in encysted dropsy, the fluid often appears as if only separated from the hand by some very thin medium, and this sensation has occasionally led to the operation of paracentesis, when no fluid has followed the introduction of the trocar." 52.

Although this disease continues, in general, through life, there are a few instances on record, where adhesions have taken place between the distended cyst, and the colon, through which a quantity of offensive fluid has passed off by stool, with ultimate recovery. In other instances, the discharge has made its way by the vagina, and could be accelerated by pressure. Even a spontaneous rupture of the abdominal parietes at the umbilicus has given exit to the contents of an encysted dropsy. Various sudden and mysterious terminations of this disease are recorded; but they are, at best, suspicious, and, at all events, so much out of the ordinary course of Nature, that they cannot be taken into account in practice.

### SCIRRHUS OF THE OVARIUM.

This is a vague term applied to various diseased states of the ovarium. In a comprehensive meaning it represents equally the degeneration of the ovarium by age, and its enlargement by the deposition of any solid structure. It is often applied to that form of ovarian disease in which a portion of the tumour is solid, and a portion made up of cysts filled with various kinds of secretions. Dr. Seymour here restricts the term to that form of the disease characterized by Dr. Baillie in the following words:—

"The ovarium (says he) is much enlarged in size, and consists of a very solid substance, intersected by membranes, which run in various directions. It resembles exactly in its texture the tumours which grow from the outside of the uterus, and I believe has very little tendency to inflame or suppurate." 58.

The ovaria are rarely affected in this way. A very remarkable specimen fell under the notice of Dr. Robert Lee, one of the most industrious and rising physician-accoucheurs of this metropolis.

"*August 9th*, 1828.—'At Blandford Mews I opened the body of a woman upwards of seventy years of age, who had died, after long suffering, from a tumour in the hypogastrium, with ascites. An induration was first perceived in the abdomen, between the navel and right ilium, nine years ago, after she had suffered considerably for some months from sense of weight and dull pain in this situation. The size of the tumour gradually increased, and about eight years ago (the belly being greatly distended with fluid,) the operation of paracentesis abdominis was performed by Mr. Blagden, and several pints of water were drawn off. In the course of the succeeding years the operation was frequently repeated; but the quantity of fluid evacuated gradually diminished, whilst the large indurated moveable mass came to occupy the whole of the lower part of the abdomen. She sunk gradually, from the interruption to the circulation caused by the tumour.'

"*Sectio Cadaveris.*—'On opening the abdomen there was found attached to the fundus uteri, on the right side, an ovarian tumour, weighing seven pounds, of a dense and fibrous structure. Several large cysts, containing a fluid varying in colour and consistence, adhered to the upper surface of the tumour. The peritoneum, in contact with its anterior surface, was converted into a cartilaginous substance, about a quarter of an inch in thickness. In the proper tissue of the uterus, at its fundus, was observed a fibro-cartilaginous tumour, about the size of a large orange. In other respects the uterus was healthy." 59.

These scirrhous tumours are said never to ulcerate, though continental pathologists frequently apply the term cancerous ulceration to them. There is, however, in the College of Physicians, a specimen of this rare disease preserved by Dr. Baillie. It is a section of a scirrhous ovarium, resembling that of a scirrhous testicle, and beginning in various places to soften down.

### Malignant or Fungoid Disease of Ovarium.

"The formation of the next, or most complicated forms of ovarian tumour, is very difficult to explain. They consist, first, of numerous cysts, with more or less fluid contents, sometimes with bony or earthy matter contained in them; often a fatty secretion, resembling lard; sometimes penetrated with long fine hair, without bulbs; but more frequently filled with albuminous secretion of varying tenacity and colour. Sometimes these secretions resemble gruel in appearance: there is often matter like soot mixed with the fluid. At other times the secretion is the colour of mahogany, from admixture of blood; and not unfrequently the liquid evacuated from one of these cysts, by the trocar, resembles, in consistence and colour, the medicine well known under the name of Griffith's mixture.

"2dly. A single large cyst springs from the ovarium, and contains within it tumours varying from the size of a pin's head to that of an orange. Sometimes the great portion of the parietes of the cyst consists of tumours growing between the external and internal, or secreting coat, the interior of the cyst having the tumours projecting into it, being filled with fluid secreted from the serous lining. The tumours, when cut into, present a semi-fluid gelatinous substance, with white bands running through it, between which bands are smaller cysts, containing the same viscid glue-like matter." 61.

The generative organs are peculiarly liable to the latter form of malignant disease. The testicles in man, the mammæ and ovaria in women, are its frequent seats. These malignant forms generally make a rapid progress—seldom lasting more than a few years at most—often terminating fatally in a few months. Cancer of the stomach is more slow than fungoid disease of the ovaria. The existence of this terrible complaint may be known in the living body by the want of nutrition and broken health of the patient—the unevenness and rapid growth of the tumour—the simultaneous enlargement of glands in other parts of the body—and the occasional occurrence of lancinating pains in the swelling. The pulse is usually quick and feeble—hectic fever arises as the disease advances—and there is an inexpressible sense of debility.

Our author here adverts to the pathological views of Drs. Baron and Hodgkin, in respect to the formation of these tumours.

"Dr. Baron, following some rather indistinct views brought forward by Boerhaave and De Haen, conceived that the tumours we have just been describing were hydatids, whose contents became more or less inspissated by time, and whose coats underwent changes of different degrees of density, from simple thickening to cartilage. The contents became coloured also, by the rupture of blood-vessels: and, by this simple view, he accounted for all the various secretions with which these tumours were found filled. For the sake of avoiding argument as to the independent life of hydatids—argument quite unnecessary, as Dr. Baron thinks, to the pathological reasoning—in his last publication he has substituted the word vesicle in their place, as being liable to no such cavil.

"Dr. Baron ascribes the formation of these vesicles to a change in the lymphatics of the part; the extremity of a lymphatic being closed, and thus forming, when distended with fluid, a pyriform vesicle, or the vesicle being formed at the intersection of numerous lymphatic vessels; of course this latter occurs oftener in the parenchyma of a viscus than on the surface. He applies this reasoning in detail, to account for the formation of malignant tumours in every part of the body. A practical observation is derived from the experiments of Dr. Baron, which may lead to important results,—that what we call malignant disease (cancer, fungus hæmatodes, medullary tubercles), may be produced in any animal by bad nutrition, arising from bad air and confinement. These conclusions of Dr. Baron can be strongly corroborated by my own experience. In the course of last summer, I was employed in dissecting several animals which died in the menageries of this city, principally with a view to the physiological observations in the first chapter. Almost without exception animals of the classes mammalia and birds died of tuberculous disease, affecting all the viscera of the body. The tubercles were principally of the kind which we call tubera circumscripta, and which have received in the French school the name of 'encephaloides,' and are found often affecting internal viscera, when cancer affects the glands or extremities. Seclusion in close cages, bad ventilation, and a want of their natural food, had produced this result. Does not this lead to the conclusion, that free air and nutritious diet, with an approximation to natural habits, is the course most likely to save those who are attacked, among our own species, by tuberculous disease?

"Dr. Hodgkin's views, that encysted tumours of the ovarium, as well as malignant tumours, arise from the development of serous cysts, have a considerable similarity to those of Dr. Baron. Dr. Hodgkin's labours are not yet entirely before the public; it is therefore improper to comment long on them. They are well worthy, and will doubtless receive, the attention of the profession. Dr. Hodgkin, as far as our present subject is concerned, conceives that a large cyst, which he calls the superior cyst, is first formed, from the inside of which tumours grow, of different sizes and shapes, pushing up the internal membrane of the superior cyst, which is reflected over them, as the pericardium and pleura are in the natural cavities of the body, lined with serous membranes. These secondary cysts contain smaller. Sometimes these smaller grow so fast as to strangulate one another, and the death of some of them causes altered appearances in the secretions of the parts. Sometimes they burst through the reflected membrane, and present a fungoid and fringed appearance, which may be seen in preparations in most collections of morbid anatomy.

"These views are very clearly and scientifically expressed in Dr. Hodgkin's paper: they do not, however, go to the extent of explaining the constitutional origin of the disease. In this respect Dr. Baron has gone further, referring these changes to disease in the absorbent system." 66.

Dr. Seymour next details some cases of this malady—some of which had been under his own care, others in the hands of his friends or acquaintances. The cases are illustrated by beautiful plates.

We shall glance at two or three of these cases.

*Case* 1. This was a lady, æt. 30, who was delivered of her third child in June, 1827. In the following September she first perceived a tumour in the left hypochondrium, unaccompanied by pain or inconvenience. Suddenly, in November, the whole abdomen became distended—and, on examination, a solid tumour was found to occupy the whole left side of that region, while a considerable fluctuation was perceptible in the right. The patient was unable to lie down in bed, or on either side. The pulse was quick and feeble—there were evening accessions of fever, followed by profuse perspirations—scanty urine—total loss of appetite and sleep. Many eminent physicians and surgeons of London visited the patient, and many different opinions were given. The spleen was suspected. Paracentesis was performed, and 22 pints of ropy fluid were drawn off, with considerable temporary relief. The operation was obliged to be repeated month after month, the solid structure constantly increasing in size. One of the incisions was burst open by the distending force ab interno, and two or three pints of a puriform fluid daily escaped, with temporary alleviation of suffering. But the patient was worn out, and died in the succeeding May.

DISSECTION.

"A large tumour occupied the place of the left ovarium, and filled the cavity of the pelvis, and great part of that of the abdomen. It was completely adherent to the front and left side of the abdominal parietes, and to the back part also on the left side, nearly as far as the vertebræ, the muscles being very thin, and having partly begun to assume the same appearance of malignant disease which the tumour itself possessed. Great part of the tumour was solid, being composed, for the most part, of transparent white gelatinous substance, with membranous partitions, containing a number of globular cysts filled with the same jelly; some others with thin transparent fluid; and one or two portions of the tumours being yellow, and harder in consistence. The greater part of the solid tumour was situated on the left side, close to the parietes, and extended from the pelvis to the ribs: but masses of the same appearance, and varying in size from that of an orange to a pea, were scattered around the principal cavity which had been tapped, and which was filled with thick purulent fluid. The whole of the external surface of the cyst and of the tumours was smooth and uniform; but the internal surface was very irregular, from the projection of these numerous globular portions of tumour into the interior of the cavity; and this internal surface was in a very vascular state, while sections of the tumours exhibited very few vessels. The inflamed appearance of the principal cavity was much greater than is usually met with in the malignant disease of the ovarium.

"The peritoneal surface of the cyst, and that of the contiguous intestines, were much inflamed, and covered with masses of recent lymph, and the cavity of the peritoneum contained a few ounces of serum; but, except at the lower part, and where it was thus in contact with the cyst, the inflammatory appearance of the peritoneum was inconsiderable. The abdominal viscera were raised by the tumour high within the chest, and pushed across to the right side and upper part of the abdomen, but were otherwise healthy. The right ovarium was much enlarged and hardened, but did not present any appearance of malignant disease." 70.

The next case was one for which our author is indebted to Dr. Henry Davies.

*Case* 2. "In October 1828, Dr. Davies was called to see Mrs. J. æt. 45, of af ull habit, sallow complexion, complaining of violent pain across the loins, with copious watery discharge from the vagina. These complaints had existed eight months; but she had felt a degree of uneasiness in the region of the uterus nearly four years. The catamenia had ceased a year before the present visit; bowels regular, pulse 76 to 80, urine free. On examination per vaginam, the os uteri was not tender to the touch, but the uterus was enlarged anteriorly. She was much relieved by local abstraction of blood, mild aperients, narcotics, and the tepid bath, during two months. On the 17th December she was attacked with violent and excruciating pain of the back, inferior part of the abdomen, and internal parts. The uterus was much increased in size, os uteri very sensible to the touch, and somewhat open.

"On the 28th December, and 18th of January, consultations were held on the case, the result of which was, that the uterus was enlarged either throughout its substance, or some body within distending it. The os uteri being distended, the orifice half an inch in diameter, and the cervix uteri obliterated, the tumour was not so hard as carcinoma, nor so firm as fleshy tubercle. The question then arose whether it was polypus, or medullary sarcoma?

"At the end of January the patient was seized, after an interval of tranquillity, with most excruciating pain, accompanied with violent expulsatory efforts. Several lumps were discharged from the vagina, small portions of which remaining, presented a ragged appearance, somewhat fleshy.

"After this the uterus became much diminished in size; the os uteri regained its natural state, and was by no means so sensible to the touch; but a tumour was now found, on examination, apparently external to the uterus and posterior to it, between the uterus and sacrum, in the recto-vaginal septum, rather more than an inch above the cervix uteri, of a nodulated shape, covered by the membrane of the vagina. The report on the 17th February was, that she had had one violent paroxysm since the last report; pulse 84; bowels and urine regular; discharge less offensive; pains less frequent. On the 25th the tumour posterior to the uterus was much enlarged, projecting below the os uteri, which appeared puckered. The tumour is now perceptible above the brim of the pelvis, its apex, in the left iliac and inguinal regions. The patient has lost flesh, and the complexion is still more sallow. In March the tumour was enlarged, and apparent above the brim of the pelvis, at the right groin. In June, having been to the Bank on urgent business, on her return the patient was attacked with rigor, followed by severe abdominal pain; the third day after which she died.

"*Sectio cadaveris.* The immediate cause of death was an attack of enteritis. The parietes of the abdomen were fat; and the omentum loaded with fat, and adherent, by its inferior edge on the right side, to a tumour. On removing the omentum, the intestines were found much distended with air, glued together by effusion of lymph, and about three pints of whey-coloured serous fluid in the cavity of the abdomen. The uterus was enlarged, and its fundus situated above the brim of the pelvis, in the left inguinal region: the left ovarium and fallopian tube sound; the right merged in the tumour. Under the peritoneal coat, near the fundus, several fibro-cartilaginous tumours were found, of a dense structure and yellowish colour. Occupying the right iliac and lumbar regions was a large tumour, with an irregular and lobulated surface, varying in colour from a light red to nearly black. It adhered to the caput coli and all the adjacent parts, filling nearly the whole of the pelvic cavity, passing behind the uterus, between the rectum and vagina, forming a projecting tumour in the vagina, pressing the uterus upwards and forwards towards the left side. This irregular mass, when cut into, and which appeared originally to be formed of the right ovarium, presented a great variety of appearance, of which it is difficult to convey an accurate idea. In some parts there were irregular-shaped cavities, containing a soft matter, having the appearance and consistence of brain, in some parts of gelatinous consistence: no part appeared organized or cartilaginous. When the soft matter was washed away, a large mass of fibrous matter, similar to that on the uterine surface, remained. On opening the uterus the os uteri was found entire, but soft and altered in structure: the cavity of the uterus contained a quantity of dark ash-coloured purulent fluid. The whole original texture of the uterus was diseased, a ragged fibrous substance, of fungoid growth, springing from its surface throughout. Several small fibro-cartilaginous tumours seemed growing also from its inner surface. The original fungoid growth which the uterus contained had been expelled from time to time, which afforded momentary relief from the occasionally insufferable pain which the patient endured." 74.

*Case* 3. Margaret Webb, æt. 52, was admitted into St. George's Hospital on the 11th June, 1829. She had had no evacuation from the bowels for more than a month. On examination per vaginam a tumour was found, about the size of an orange, adhering to, and external to, the upper part of the vagina, so pressing on the rectum as to render the passage of a gum elastic tube very difficult, and the ordinary administration of an enema impossible. Two months previously she was attacked with severe pains in the right hypogastric region, which still continued. The constipation had been gradually increasing for two years. The countenance was pale and sallow —pulse quick and weak—tongue furred. Mr. Babington endeavoured to return the tumour, conceiving it to be the ovary. Some adhesions gave way, and the tumour receded out of reach. One of the small cysts, however, gave way, and its contents were extravasated into the abdominal cavity. Inflammation followed, and terminated fatally.

"On opening the body, the peritoneal covering of the bowels was seen much inflamed, and the convolutions of the intestines glued together by recently effused lymph. The upper portion of the intestines were greatly distended by fæces. Opposite the commencement of the first lumbar vertebra, the great intestine was found much thickened, to the extent of nearly three inches; and at the centre of this thickened portion the cavity was so entirely obliterated that even fluid could not be made to pass through it. The internal surface of the thickened intestine was partially ulcerated. On examining the uterus, the right ovarium was found changed into a mass of soft matter, not very dissimilar to the substance of the brain. This matter, more or less fluid, was arranged in cysts; one of which having given way, in the endeavour to restore the ovarium to its natural position, had poured out its contents into the peritoneal cavity. The tumour had adhered to the posterior and inferior part of the fundus uteri, by the opposite peritoneal surfaces, thus formirg the tumour felt on examination per vaginam." 78.

We must now dedicate a few pages to the subject of treatment.

We are glad to find our talented author speak confidently on this point. "If, after considering *seriatim* the diseases of this organ, we proceed to seek for remedies for its various affections, we shall find that we possess very powerful means of subduing disease, and still more effectual ones of calming and alleviating the distress arising from an acknowledged incurable state." Dr. S. hopes, and so do we, that medical men will not give up their whole attention to the investigation of diseased structure, but allot a portion of it to the search after remedies.

We need not dwell on the treatment of acute inflammation of the ovaria, which is the same as for a similar inflammation in any other part. Local depletion and perfect quietude are the essentials. In simple encysted dropsy, the excitement of the urinary and other secretions or excretions has not the beneficial effects that are found to result in ascites or anasarca. But where general effusion into the peritoneal cavity has occurred, the increase of such secretions are useful. The infusion of pyrola umbellata is much recommended by Dr. Seymour—a pint daily. To emetics, as promoting absorption, Dr. S. seems partial, and relates some curious cases of the surprising removal of glandular swellings by the operation of vomiting. These we need not detail.

"It is obvious that the sweeping objection which would exclude bloodletting in this disease, must have arisen from misunderstanding its pathology; when accumulation of fluid or growth are proceeding rapidly, when there is a quick pulse, irregular heat of skin, and acute pain in the part, it is obvious that inflammatory action is going on within the cyst, and will probably eventually be extended to the neighbouring peritoneum; the fluid secreted is mixed with shreds of lymph, or thickened by the diffusion of purulent matter; under such circumstances the use of the lancet is employed with much benefit. Even when great depression of vital power has apparently existed, the relief obtained has been very great, and similar to what is experienced in inflammation of an acute nature, when seated in other serous membranes. The pulse has risen in force and diminished in frequency under the flow of blood; the crassamentum has been unusually firm, and the buffy coat very distinct on the coagulated blood. The oppression under which the patient laboured has vanished under the repetition of the treatment; and although the disease has been by no means cured, the strength of the patient has been saved, and she has perhaps been brought into the situation in which paracentesis may be employed without risk. It is in such cases that mercury is useful, and as in other inflammatory diseases these remedies appear to be nearly similar in their effects, one diminishing, the other altering vascular action. The comfort experienced after such loss of blood, by the administration of opium, is certainly equal to, if not greater, than that which occurs in inflammation affecting vital organs, and seems to realize the feeling and almost poetical expression of the late Dr. Currie, of Liverpool: 'The patient sinks into a sleep, which is ill exchanged for the realities of life.'" 96.

Moderate purgation, by removing flatulence and fæcal accumulations, is useful; but hypercatharsis is distressing, and may prove dangerous by breaking one or more of the cysts in the act of straining.

Morgagni, and many others since his time, appear to have entertained great reluctance towards the operation of paracentesis. But the moderns have lost all dread of this kind. Our author steers a middle course—the "auream mediocritatem"—avoiding a too early recourse to the trocar on one hand—and a too fastidious delay of surgical aid on the other, by which the patient is subjected to a painful, almost unendurable distention.

"Two methods have been proposed then for emptying the cyst, and for promoting its entire contraction.

"1. A considerable incision, in order to empty the cyst entirely of its contents, leaving in a canula or bougie, to excite contraction of the cyst, and prevent the re-collection of fluid.

"2. Injections into the cyst.

"For the first method of practice it has been urged, that operations on the abdomen, although dangerous, are by no means fatal; and the cyst often containing matters of various tenacity, these contents will not escape through an ordinary canula.

"A very remarkable instance of the application of this practice, and a very strong proof of the impunity with which operations conducted with considerable roughness may sometimes be successful, is contained in the 33d vol. of the Philosophical Transactions, by Dr. Houston, more than a century ago. This was the case in a woman, æt. 58, of an ovarian tumour of 13 years duration. I subjoin the account of the operation in his own words:—

"'The operation of puncturing the abdomen being proposed, she consented. Accordingly, with an imposthume lancet, I laid open about an inch; but finding nothing issue, I enlarged it two inches, and even then came nothing forth but a little thin yellowish serum, so I ventured to lay it open about two inches more. I was not a little startled, after so large an aperture, to find only a glutinous substance bung up the orifice. The difficulty was, however, to remove it. I tried my probe, and endeavoured with my fingers, but all in vain; it was so slippery that it eluded every touch, and the strongest hold I could take.

"'I wanted in this place almost every thing necessary, but bethought me of a very odd instrument, yet as good as the best in its consequence, because it answered the end proposed. I took a strong fir splinter, such as the poor in that country use to burn instead of candles; I wrapped about the end of the splinter some loose lint, and thrust it into the wound; and by turning and winding it, I drew out above two yards in length of a substance thicker than jelly, or rather like glue fresh made and hung out to dry; its breadth was about ten inches. This was followed by nine full quarts of such matter as is met with in steatomatous and atheromatous tumours, with several hydatids, of various sizes, containing a yellowish serum, the least of them larger than an orange, with several large pieces of membranes, which seemed to be parts of the distended ovary. I then squeezed out all I could, and stitched up the wound in three places.'

"This patient recovered, and lived fourteen years afterwards, without any return of the disease." 101.

The next operation of this kind is recorded in the memoirs of the Royal Academy of Surgery, Paris. The celebrated Le Dran was the operator. He made an incision into the tumour, and left the trocar in the wound, through which he injected mild fluids. The patient survived the operation four years, with a fistulous opening, which never entirely closed. In another similar operation the canula was left in; and, at the expiration of two years, the fistula closed, and the patient recovered. Mr. Key, of Guy's Hospital, has tried this plan in three instances, without success. The fol-lollowing are his words, in a communication to Dr. Seymour.

" 'I find notes of three cases in which the instrument was left in after tapping an encysted dropsy. The issue has not been such as to lead me to expect much from the plan. One case was favourable for the treatment, as the fluid was of the serous character. The two others contained a fluid of much thicker consistence; in one it resembled mucilage, in the other a dark coffee-ground fluid. Case 1. A strong and otherwise healthy woman, æt. 42, single. Dropsy of four years standing. Twenty-seven pints drawn off, resembling straw-coloured serum; no inflammation followed. In two months fluid again collected; tapped; and twenty-one pints of same character removed. A piece of elastic gum catheter left in, but closed; for three days pain, but not considerable; slight febrile symptoms; on the third day plug withdrawn, and a few ounces of turbid serum removed. Experienced relief. The same operation repeated on the 9th, 13th, and 18th of May. At each successive operation the fluid assumed a more turbid and inspissated character, shewing the progress of inflammation. At the last she began to complain of so much general tenderness, and so much fever excited, that I was induced to comply with her request to withdraw it. The treatment certainly retarded the formation of fluid, for I had not occasion to tap her until six months afterwards, when the fluid was found to be of the serous kind, containing a few flakes of lymph. The medical treatment consisted in mild purgative remedies.

" 'The second case is that of a female, æt. 33, having had ovarian dropsy for two years and a half; the tumour solid in some parts, with a large cyst on right side; the health impaired of late as the tumour increased. The bougie was introduced after tapping; the fluid drawn off was of the mucilaginous kind, of a light brown colour. On the third day she complained of great pain across the scrobic. cordis, which was relieved by fomentations. On fifth day, pain returning, with sickness and a febrile pulse, I thought it advisable to take out the bougie. The fluid again collected after a short interval, and was removed; it retained the same character. This patient died out of the hospital in a year after; and, on inspection, the ovarian tumour was divided into several cysts of various sizes, with tense fibrous septa.

" 'The other case was a delicate young married woman, without children, exceedingly florid complexion, and of but little constitutional power. The fluid was of a dark reddish coffee-ground color, about seventeen pints in quantity. A piece of elastic catheter was left in after the operation; obliged to be withdrawn on the following day, in consequence of the severe constitutional irritation which followed. The fever and tenderness of the belly increased for four days, and an abscess formed between the peritoneum and integuments, which burst at the opening made by the trocar. Under the continued suppuration she sank; and not being allowed to inspect her, we could not ascertain if the abscess communicated with the cyst; of this, however, we had strong suspicion.' " 105.

In the true scirrhus of the ovarium our author is unable to point out any remedy that can be relied on. Mercury, iodine, the caustic alkali, conium, and muriate of lime, have all been employed for the removal of morbid growths—but not with very great success. Dr. Seymour descants on the power of mercury in dissolving adhesions resulting from inflammatory action, and then adverts to dropsy, as so often dependent on disorganization of the heart, liver, lungs, or kidneys.

"If this disorganization has long existed, if the inflammatory action which produced it has entirely ceased, then perhaps will the employment of mercury be found worse than useless, diuretics and purgatives, according to the circumstances of the case, affording the best chance of relief; but if the case be recent, and inflammation, in a more or less acute form, is going on, many a patient has his life prolonged by the administration of mercury, combined with venesection and the use of the salts of potass." 108.

On these principles the beneficial employment of mercury is limited to those cases in which vascular excitement has immediately preceded the enlargement, and still continues, in which case its growth may be entirely stopped, and the already formed increase of bulk diminished.

"It must be remembered also, that ovarian tumours sometimes increase rapidly, and by pressing on neighbouring parts produce inflammation of surrounding textures; where such inflammation ensues, the employment of mercury will be found useful, for at this time the blood drawn will be found covered with buffy coat. The membrane which lines these cysts we have seen is nearly allied in structure to the natural serous membranes of the body; it likewise is often attacked by inflammation after tapping, or from external injury. Here again mercury is useful, and will restrain even more powerfully than venesection the progress of the mischief." 109.

Excepting under the foregoing circumstances, Dr. S. thinks that mercury can be of little or no use—perhaps of disuse. Dr. S. then takes up the consideration of iodine. After adverting to the injurious effects of this medicine, when incautiously administered, he relates the following case communicated to him by Mr. Brodie.

" 'I have employed iodine as an internal medicine in a great number of cases of morbid growth, without any manifest effect arising from its exhibition. In two cases, however, and in two only, it was productive of the greatest benefit, effecting that which I could scarcely have supposed that any medicine was able to accomplish.

" 'In one of these cases, which I attended with Mr. Pennington, the patient laboured under a tumour on one side of the tongue, and imbedded in its substance, of about the size of a nutmeg, of an irregular form, hard to the touch, and having a well-defined margin. The disease had existed between one and two years, gradually making progress; and it had resisted the internal use of arsenic, as well as a course of sarsaparilla, combined with oxymuriate of mercury. As the surface of the tongue was furred, and there were some other symptoms which seemed to indicate a deranged state of the digestive organs, we prescribed, in the first place, the pilula hydrargyri, with a gentle aperient, and a light bitter with soda. Under this treatment the tongue became clean, but there was no perceptible alteration in the local disease. We then administered the tincture of iodine three times daily in moderate doses, gradually increased. In a fortnight the tumour was evidently smaller, and at the expiration of about eight weeks it had nearly disappeared. The patient was sent into the country, being directed to continue the use of the iodine for some time longer. This was upwards of four years ago, and I have not seen the patient since; but I have been informed that the cure is complete.

" 'The second case was that of a man who was admitted into St. George's Hospital on account of a tumour, situated on one side a little below the axilla. It was of the size of a small orange, unattended by pain, and bearing no other marks of inflammation, and quite moveable beneath the skin. Having removed it by the knife, I found, on making a section of the tumour, that it was composed of a brown solid substance, of a firmer consistence, and to all appearance more highly organized than fungus hæmatodes, and of an uniform structure throughout, except that externally it was covered by a thin membranous cyst closely adhering to it. Sometime afterwards the same man applied at the hospital a second time, having two tumours on the neck, each of the size of a double walnut. These bore no resemblance to the common enlarged glands which occur in this situation, and so exactly resembled that which had been removed from the side, that no one entertained a doubt as to their being exactly of the same nature.

" 'Conceiving that there were some obvious objections to a second operation for the removal of a disease, so manifestly depending on a constitutional cause, and knowing nothing better to be done, I prescribed the tincture of iodine to be taken internally. Under this course of treatment, which was continued for several weeks, the tumours gradually diminished in size, and ultimately disappeared. I have heard nothing of the patient since; but as I told him that he should be received into the hospital again whenever he applied for that purpose, I think that in all probability he has had no return of his complaint.

" 'I have no right to say, that in these cases the tumours were of a malignant nature; at any rate, they were not malignant tumours of the worst kind. I have, however, exhibited the tincture of iodine in many cases of truly malignant disease, and in a few instances, as it appeared, not without some temporary advantage. For example, I was consulted concerning a lady who was supposed to labour under a tumour of the breast. I found, however, on examination, that the breast itself was in a healthy state; and that in this, as in some other cases which have fallen under my observation, the apparent enlargement of the breast was the consequence of its being elevated by a tumour beneath it The tincture of iodine was given internally, and under its use the tumour became so much reduced in size, that I had the credit with the patient and her friends of having cured an obstinate disease. The amendment, however, was of short duration. Soon after discontinuance of the medicine, the tumour began again to increase in size; and the iodine, which was a second time administered, had now no dominion over it. The patient ultimately died; and on inspecting the body, it was ascertained that there was a medullary or fungus tumour, which had its origin in one of the ribs below the breast and pectoral muscles. The same disease existed also in other parts of the body.' " 115.

A remarkable case is next detailed by Dr. Seymour himself. A female, aged 31, was admitted into the Asylum of Health, under Dr. Badeley, in March, 1827. A large tumour could be traced into the pelvis, and occupying the whole right side of the abdomen. It was hard to the touch, irregular, and conveyed an obscure sense of fluctuation. It had existed 18 months. The health was tolerably good. The tincture of iodine was exhibited for two months, gradually increasing the dose to 20 drops twice a day, with external frictions of the same. The tumour appeared to grow gradually smaller, and, at length very violent constitutional symptoms came on, viz. tremblings, great distress of mind, and lowness of spirits, to which succeeded signs of internal suppuration—a very quick pulse, brown tongue, rigors, profuse sweats. At the expiration of a fortnight the patient began to pass purulent matter by the rectum and vagina, extremely fetid. This discharge continued for several weeks. She was now allowed generous diet and tonics. She was sent into the country, and returned in five weeks, her strength restored, and the tumour nearly gone. Six months after this she was examined by a celebrated physician-accoucheur, who could discover no tumour. We believe, however, that this patient is now in St. George's Hospital in a very deplorable state.

Before quitting the subject of iodine, we ought to advert to the treatment employed and recommended by Dr. A. T. Thompson. This is, the evacuation of the fluid from the cells by tapping, and then the steady administration of iodine. We believe it will be found that this potent medicine has a strong diuretic effect—at least we have seen this effect produced by it in a very marked manner, in ascites with enlarged liver.

"The third of the remedies which have enjoyed a high reputation as a deobstruent is liquor-potassæ. This medicine, employed in as large doses as the stomach will bear, appears to have been successful in discussing indolent scrofulous tumours, and those of a steatomatous kind. It is with diffidence that I offer any result of my own experience; but in diseases of a malignant nature, affecting internal parts, it has appeared to me to produce more alleviation than any other remedy with which I am acquainted. This applies, however, principally to those tumours when they are not attended with acute pain, or any considerable symptomatic fever.

"Liquor-potassæ has been recommended in ovarian disease of the kind we are considering, and the general health appears often to have been greatly improved during its use; and the formidable disease itself is reported to have disappeared under its employment.

"The liquor-potassæ, in such cases, appears to act by inducing suppuration in the cysts, which is afterwards discharged after adhesions formed with neighbouring viscera. In this respect its action resembles that of iodine, and is contra-indicated when increased vascular action is present; hence it would appear to be most useful in those cases to which mercury is inapplicable; and, in fact, it is in the leucophlegmatic habit of body that it appears to be most beneficial, whether as a curative or only as a palliative agent.

"Dr. Warren has favored me with the account of a case which occurred under his care several years ago, in which this remedy was employed in very large doses, as large as the stomach could bear it, at short intervals. After some weeks, softening of the tumour took place, adhesion with the great intestine, an opening was formed, and much purulent matter, united with other secretions of various consistence, such as are observed in these tumours, passed by stool. The swelling subsided, and the patient entirely recovered her health." 119.

Mr. Abernethy has strongly recommended a series of blisters, after the fluid has been drawn off, as a preventive of its re-accumulation. The muriate of lime has been much lauded by Dr. James Hamilton, of Edinburgh, in conjunction with "percussion of the tumour."

"'Adverting (says he) to the effects of percussion and of pressure in chronic rheumatism, and knowing the influence of the continued use of the muriate of lime in indolent glandular swellings, the author was led to the trial of those several means, as being at any rate perfectly safe. He advised, therefore, that moderate and equable pressure of the abdomen should be made, by means of a suitable bandage; that the enlarged part should be subjected twice a day to gentle percussion; and that a course of small doses of the muriate of lime should be continued for at least several months. Where pain or tenderness was experienced on the ovary being pressed upon, he recommended, in addition to the above means, the daily use of the warm bath.

" 'This plan of treatment has been much more successful than he had anticipated. In seven cases in which it was tried, the enlargement has so completely subsided that it is no longer tangible. There could be no mistake in the majority of these cases, not only because the size of the diseased ovary was very considerable, the fluctuation was distinct, and all the ordinary characteristics well marked, but also because the nature of the affection had been previously ascertained by the most experienced practitioners in London.

" 'In the first three cases the author considered that there might be some accidental coincidence independent of the remedies employed, and therefore he did not venture to allude to them even in lecturing, being always unwilling to give any hints which might lead to delusive speculations in the practice of physic; but the fortunate issue of four additional cases entitles him to presume that the above means of cure bid fair to prove extensively useful.' " 123.

The last measure—the ANCEPS REMEDIUM—is extirpation of the whole tumour. We have laid before our readers, on former occasions, all that is known of this formidable operation. We need not here reiterate them. The operation certainly has been successful, both in this country and on the Continent; but we are inclined to agree with the sensible author of the work under review, that—" the arguments against such an operation are numerous and strong, while the probabilities of success are very small."

We think that both the author and the reader will acknowledge that we have done ample justice to the work, by a faithful and extended analysis of its contents—an analysis that will travel through every region of the globe, and consequently experience a diffusion which the original can never hope to attain.

